# Identification of a variant-specific phosphorylation of TH2A during spermiogenesis

**DOI:** 10.1038/srep46228

**Published:** 2017-04-07

**Authors:** Masashi Hada, Koji Masuda, Kosuke Yamaguchi, Katsuhiko Shirahige, Yuki Okada

**Affiliations:** 1 Laboratory of Pathology and Development, Research Center for Epigenetic Disease, Institute of Molecular and Cellular Biosciences, The University of Tokyo, 1-1-1 Yayoi, Bunkyo, Tokyo 113-0032, Japan; 2 Graduate School of Agricultural and Life Sciences, The University of Tokyo, 1-1-1 Yayoi, Bunkyo, Tokyo 113-0032, Japan; 3 Laboratory of genome structure and function, Research Center for Epigenetic Disease, Institute of Molecular and Cellular Biosciences, The University of Tokyo, 1-1-1 Yayoi, Bunkyo, Tokyo 113-0032, Japan; 4 Graduate School of Art and Sciences, The University of Tokyo, 3-8-1 Komaba, Meguro, Tokyo 153-8902, Japan

## Abstract

Tissue-specific histone variant incorporation into chromatin plays dynamic and important roles in tissue development. Testis is one such tissue, and a number of testis-specific histone variants are expressed that have unique roles. While it is expected that such variants acquire post-transcriptional modifications to be functional, identification of variant-specific histone modifications is challenging because of the high similarity of amino acid sequences between canonical and variant versions. Here we identified a novel phosphorylation on TH2A, a germ cell-specific histone H2A variant. TH2A-Thr127 is unique to the variant and phosphorylated concomitant with chromatin condensation including spermiogenesis and early embryonic mitosis. In sperm chromatin, phosphorylated TH2A-Thr127 (=pTH2A) is co-localized with H3.3 at transcriptional starting sites of the genome, and subsequently becomes absent from the paternal genome upon fertilization. Notably, pTH2A is recurrent and accumulated in the pericentromeric heterochromatin of both paternal and maternal chromosomes in the first mitosis of embryos, suggesting its unique regulation during spermiogenesis and early embryogenesis.

Histone variants contribute to dynamic epigenetic changes by replacing their canonical counterparts in a wide range of biological processes. Germ cell-specific histone variants are one of the most diverse groups^1^,^2^,^3^,^4^. Thus far, more than 10 such variants have been identified^5^,^6^, and most of these are involved in germ cell-specific functions such as meiosis and sperm chromatin condensation. Compared to histone variants expressed in somatic cells, the function of germ cell-specific variants has been poorly characterized for a variety of reasons, including the lower abundance of variants compared to their canonical counterparts, the difficulty associated with generating specific antibodies, and the lack of proper cell culture systems.

The germ cell-specific histone variants TH2A (Hist1h2aa) and TH2B (Hist1h2ba) were originally identified in rat as testis-specific counterparts for canonical H2A and H2B^7^,^8^, respectively. The presence of TH2A has been reported in mouse and rat with 92.9% amino acid sequence similarity, and their human forms have been recently identified^7^,^8^,^9^,^10^ (Fig. S1a). In all of these species, the C-terminal (aa.123~) is the most distinctive difference to canonical H2A. *Th2a* and *Th2b* genes are adjacently encoded in Chr. 13 (mouse), Chr. 17 (rat), and Chr. 6 (human), and bi-directionally transcribed from the same promoter during S phase^10^. This is because both contain the consensus sequence element in 3′ UTR, which is involved in the S phase-specific stabilization of their mRNAs^10^,^11^. The proteins are translated and persist in meiosis, where they replace around 60% of their canonical forms^12^. A recent study has demonstrated that both variants are also expressed in oocytes and fertilized early embryos^13^. Loss-of-function studies using *Th2a/Th2b*-deficient mice have demonstrated that maternal TH2A/TH2B is dispensable for oogenesis, while it contributes to the activation of the paternal genome after fertilization, suggesting a crucial role in transcription^13^. In contrast, *Th2a/Th2b*-deficient male mice exhibit impaired release of cohesin during meiosis and failure of proper histone removal during spermiogenesis^14^. Although single loss-of-function analyses have not been reported for TH2A, a study of Th2b-deficient mice has indicated that TH2B is dispensable for spermatogenesis and fertility^15^. This suggests that the impaired spermatogenesis observed in *Th2a/Th2b*-deficient males is likely caused by the absence of TH2A, or by the synergistic effect of both absences. In addition, it is noteworthy that exogenous overexpression of TH2A, TH2B, and the histone chaperone nucleoplasmin 2 in somatic cells enhances reprogramming by stem cell-specific transcription factors (OCT4, SOX2, KLF4, and c-Myc). It does so by modulating chromatin structure in both mouse and human^13^,^16^, implying the importance of investigating these histone variants.

Among the different types of histone modifications, phosphorylation plays an important role in cell division. Phosphorylation at multiple Serine/Threonine residues (Thr 3, Ser 10, Thr 11, and Ser 28) in H3, and at Thr 120 in H2A, in particular is highly conserved among different species^17^,^18^. Phosphorylation events play critical roles in proper chromosome segregation accompanied by chromatin condensation in both mitosis and meiosis. It does so by directly or indirectly recruiting Aurora kinase B/C, a catalytic subunit of the chromosome passenger complex that binds the centromeric nucleosome^19^,^20^.

In testis, marked chromatin condensation occurs during spermiogenesis, as the majority of histones are replaced by transition proteins and subsequently by protamines. During the nuclear elongating period, incorporation of histone variants and specific histone modifications affect the higher-order chromatin structure, and play critical roles in transcriptional regulation as well as chromatin compaction, which appears to be critical for the maintenance of genome integrity^21^,^22^. One well-characterized modification is the hyperacetylation of histone H4, which is observed in elongating spermatids just prior to histone removal, and is retained in epididymal spermatozoa^23^,^24^. It is suggested that H4 hyperacetylation leads to an open chromatin structure that facilitates histone removal^6^. H3K9 acetylation in elongating spermatids has been reported, whereas H3K9 methylation is predominantly observed in the chromocenter of round spermatids, and subsequently in the pericentromeric heterochromatin of elongating spermatids^6^,^25^. H3K4 and H3K27 methylations are also abundant in elongating spermatids^26^,^27^. A recent comprehensive mass-spectrometry analysis of male germ cells has identified various post-transcriptional modifications in canonical histones and their variants during spermatogenesis. Some of these are uniquely present in sperm but not in the previous spermatogenic stages, and are conserved between mouse and human^2^.

In the present study, we report on the identification of the new phosphorylation site threonine 127 of the histone H2A variant TH2A (=pTH2A) in mouse spermatozoa by mass spectrometry. Experiments using pTH2A-specific antibody have successfully demonstrated the dynamics of pTH2A during spermiogenesis and in fertilized embryos, and suggest that pTH2A may be involved in chromatin condensation.

## Results

### TH2A Thr127 is phosphorylated in sperm chromatin

**Figure 1:**
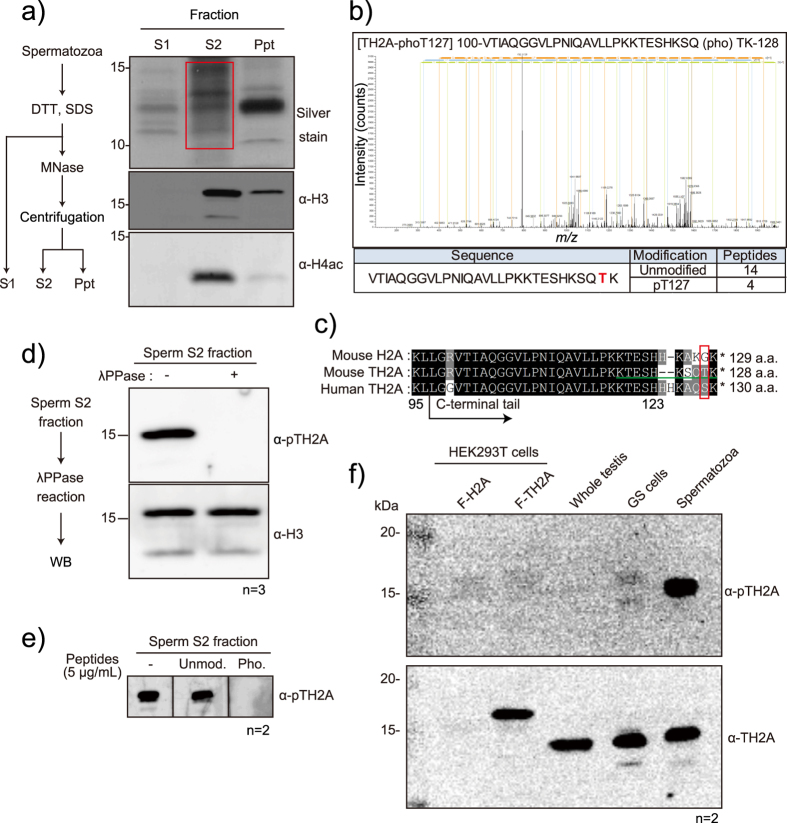
Identification of pTH2A in mouse spermatozoa. (**a**) An experimental scheme to prepare nuclear fractions from spermatozoa. Proteins are biochemically divided into three fractions (S1, S2, and Ppt). Silver staining and western blot analyses of each fraction are as indicated. The red box indicates the area to be digested and subjected to LC-MS analysis. (**b**) Representative MS/MS spectrum of pTH2A peptides (upper panel). The y-axis indicates intensity of MS/MS spectra, and the x-axis indicates *m/z*. Phosphorylated threonine is highlighted in red, and the identification number for each peptide is summarized (lower panel). (**c**) Alignment of amino acid sequences between mouse H2A, mouse TH2A, and human TH2A. Black arrow, start of the C-terminal tails; red box, the phosphorylated threonine in mouse TH2A; green line, epitope sequence for generation of the anti-pTH2A antibody; Roman numeral, number of amino acid from the N-terminus. (**d**) Western blot analysis of sperm S2 fraction treated or untreated with lambda protein phosphatase (λPPase). Experimental procedure (left panel) and representative images of western blot analysis (right panel) are indicated. (**e**) Peptide blocking assay. Indicated peptides were added to the diluted primary antibody during the reaction. (**f**) Western blot analysis of pTH2A (upper panel) and TH2A (lower panel). Protein extracts of HEK293T cells overexpressing FLAG-tagged H2A (F-H2A) and -TH2A (F-TH2A), whole testicular cells, germ stem cells (GS cells), and spermatozoa were analyzed. Number of biological replicates of each experiment is shown as n.

To identify novel histone modifications in mouse spermatozoa, we isolated caudal epididymal sperm and extracted the chromatin via a combination of dithiothreitol (DTT), sodium dodecyl sulfate (SDS), and micrococcus nuclease (MNase) treatment (Figs 1a and S5a). The solubilized fraction (S2) was resolved by SDS-PAGE (Fig. 1a), and proteins around 10–20 kilodalton, where core histones should be enriched, were subjected to liquid chromatography-mass spectrometry (LC-MS). This analysis revealed acetylation on the histones H4-K5, -K8, -K12 and -K16, and H4-K20 mono-methylation (Fig. S1b,c), which are consistent with previous reports^28^. Interestingly, a phosphorylation site at Thr127 of TH2A (TH2A-pT127 or pTH2A), which is located at the end of C-terminus of TH2A and not conserved in canonical H2A (Fig. 1b,c), was also identified. Although the C-terminal sequence of human TH2A is not identical to that of mice, the amino acid corresponding to mouse Thr127 is Ser129 in human and surrounding sequences are well-conserved (Fig. 1c).

To gain insight into the *in vivo* function of this phosphorylation, we generated an antibody against pTH2A. Two batches of antibody from different rabbits both specifically recognized the Thr127-phosphorylated peptides of TH2A but not the unmodified peptides by enzyme-linked immune sorbent assay (ELISA) (Fig. S2a–d). This suggests that they specifically recognize Thr127-phosphorylated TH2A. Western blot analysis further demonstrated that the antibody could recognize a band corresponding to the size of TH2A from the sperm histone extract, but not any other bands (Figs 1d and S5b). In addition, the band disappeared after phosphatase treatment (Figs 1d and S5b), further supporting its specificity. Furthermore, the signal also disappeared when competed with a blocking peptide (Figs 1e and S5c). These data strongly suggest that our antibody specifically recognizes pTH2A.

We next performed western blot analyses to see whether pTH2A was detected in testicular extract. We found that pTH2A was highly enriched in caudal epididymal spermatozoa, whereas it was below the detection level in the nuclear extracts prepared from whole testis and *in vitro*-cultured germ stem cells (GS cells), respectively. Notably, TH2A itself was however expressed at equivalent levels in these cell types (Fig. 1f, lane 3–5, and S5d). Moreover, Flag-tagged TH2A that was overexpressed in HEK293T cells was not recognized by the anti-pTH2A antibody (Fig. 1f, lane 2, and S5d), suggesting the possibility of cell type-specific phosphorylation of TH2A.

### TH2A is phosphorylated in the final stages of spermatogenesis

**Figure 2:**
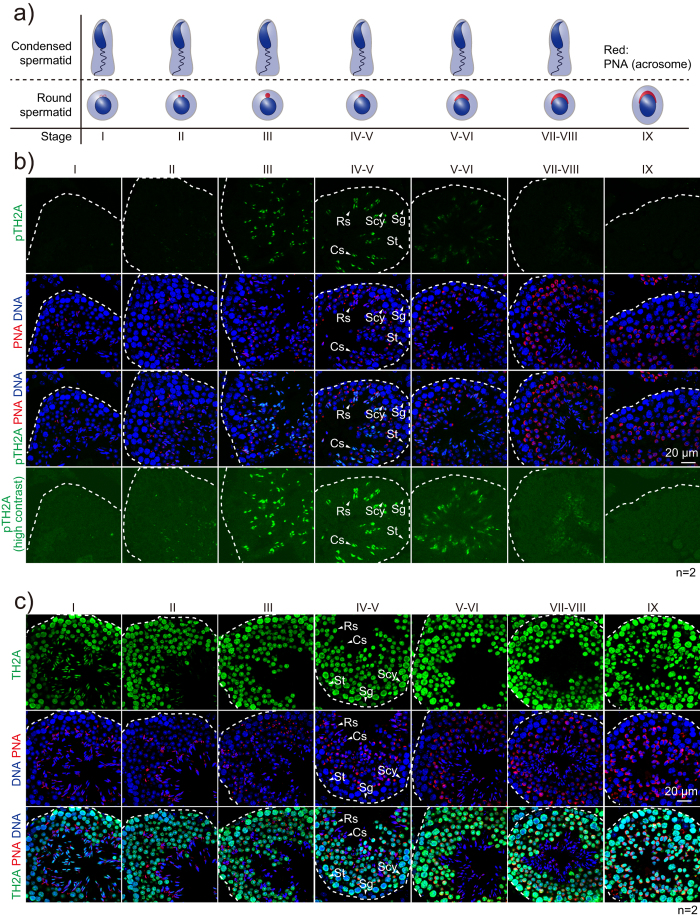
TH2A is phosphorylated in condensed spermatids. (**a**) Scheme of spermiogenesis from Stage I to IX. Stages of each seminiferous tubule are determined by the staining pattern of PNA (red). Roman numerals indicate the spermatogenic stages of the seminiferous tubule. (**b**,**c**) Immunofluorescent staining of testicular tissue against pTH2A (**b**) and TH2A (**c**). Roman numerals indicate the spermatogenic stages of the seminiferous tubule. Representative testicular cells are marked by white arrowhead at spermatogenic stage IV to V seminiferous tubules. Antibodies used and scale bars are as indicated. Number of biological replicates of each experiment is shown as n. Abbreviation: St, Sertoli cell; Sg, spermatogonium; Scy, spermatocyte; Rs, round spermatid; Cs, condensed spermatid.

The failure to detect pTH2A signal in the whole testis extract (Fig. 1f) prompted us to perform immunostaining to examine whether testicular germ cells possess pTH2A, or if it only exists in the caudal epididymal (i.e. mature) spermatozoa. Peanut agglutinin (PNA), which binds to the glycosylated proteins of the acrosomal region of post-meiotic germ cells including mature sperm^29^,^30^, was co-stained to determine the spermatogenic stages (Fig. 2a). In the seminiferous tubules, germ cells are aligned as layers, in which the most primitive cells are localized in the basal side, and they move inside of the lumen upon differentiation. As a result, condensed spermatids are localized in the most inside of the tubules, and the spermatogenic stages can be distinguished by staining pattern of PNA in round spermatids, which are adjacent to condensed spermatids (Fig. 2a). The immunostaining indicated that pTH2A started to appear in the condensed spermatids of Stage III seminiferous tubules, in which protamine incorporation and nuclear chromatin condensation were almost completed^31^, and it became more intense in Stage IV-V (Figs 2a,b and S3b–d). TH2A itself was ubiquitously expressed in all germ cell stages including spermatogonia, spermatocytes, and spermatids, but not Sertoli cells (Fig. 2c and S3b). In the spermatids of the last spermiogenic step in Stage VII-VIII seminiferous tubules, the immunofluorescent intensity of pTH2A was reduced, presumably due to the difficulty of the antibody in accessing the highly-condensed sperm chromatin rather than the dephosphorylation (Fig. 2a,b). Finally, the spermatid signal was diminished after sperm release in Stage IX (Fig. 2a,b). None of these signals were observed in IgG-control sections (Fig. S3a,b). Together, these results suggest that pTH2A occurs at the final stages of spermatogenesis, when nuclear events such as transcription are generally shut down due to highly condensed chromatin structure. In addition, the failure of detecting pTH2A in whole testis extract by western blotting (Fig. 1f) is likely because the relative amount of pTH2A in the extract was insufficient to be detected.

### pTH2A is accumulated at transcriptional starting sites of sperm chromatin

**Figure 3:**
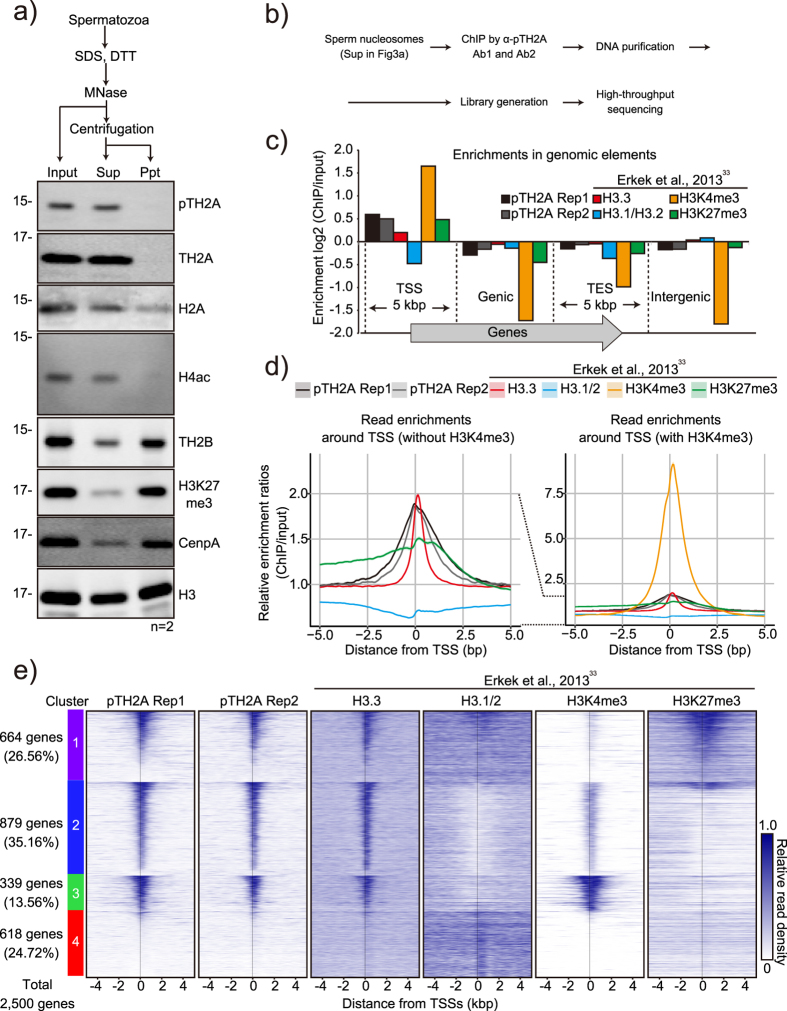
pTH2A is enriched in TSSs of sperm genome. (**a**) Western blot analysis of sperm chromatin fractionated by MNase digestion. Antibodies used to detect each histone variant and modification are indicated. Number of biological replicates is shown as n. (**b**) An experimental procedure for ChIP-seq analysis using sperm nucleosome. (**c**) Enrichment of pTH2A replication 1 (Rep1), pTH2A replication 2 (Rep2), H3.3, H3.1/2, H3K4me3, and H3K27me3 in genomic elements. The mouse genome was divided into four elements: TSS region, 5 kbp around TSSs of all RefSeq genes; TES region, 5 kbp around transcriptional end sites (TES); genic region, region between TSS and TES; intergenic region, regions other than the former three elements. Data sets of H3.3, H3.1/2, H3K4me3, and H3K27me3 as well as their controls were obtained from Erkek *et al*.^33^. Enrichments (ChIP/input) are shown in log2. (**d**) Average profiles of each histone variant and modification enrichment ±5 kbp around TSS. H3K4me3 is excluded in the left panel to more clearly show other histone profiles. Ribbon: 95% confidence interval. (**e**) Heat map of ChIP-seq read density of each histone around TSSs. 2,500 genes were randomly selected and clustered into four groups: cluster1, 26.56% (664 genes); cluster2, 35.16% (879 genes); cluster3, 13.56% (339 genes); cluster4, 24.72% (618 genes). Genes are sorted by the average read density of pTH2A rep1 around TSSs (±1 kbp). Each row represents a gene centered on the TSSs. Relative read density denotes the scaled read density of each ChIP-seq data.

The unique behavior of pTH2A, which is acquired in the very last step of spermiogenesis, may suggest its specific roles in mature sperm and/or fertilized embryos. Therefore, we next investigated where pTH2A occurred in the sperm genome, and performed chromatin immunoprecipitation-sequencing (ChIP-seq) analysis. Biochemical solubilization of sperm chromatin was performed by treatment with a high concentration of DTT and MNase based on previous publications^32^,^33^ (also see Methods), followed by fractionation by centrifugation (Fig. 3a). Western blot analyses demonstrated that both pTH2A and TH2A, as well as acetylated H4, were enriched in the supernatant, while TH2B, H3K27me3 and CENP-A were mainly observed in the pellet (Figs 3a and S5e). This suggests that TH2A and TH2B possess distinct biochemical properties in sperm, and that TH2A-containing nucleosomes are more sensitive to MNase treatment. Canonical H2A exhibited modest enrichment in the supernatant but was also retained in the pellet (Fig. 3a). The supernatant fraction was subjected to ChIP-seq analyses using anti-pTH2A antibody to further identify the localization of pTH2A in the sperm genome (Fig. 3b). To assure the specificity of our antibody and the reproducibility of the experiments, both antibody #1 (Ab1, Rep 2) and #2 (Ab2, Rep 1), obtained from different rabbits, were used as replicates for immunoprecipitation (Fig. S2a–d). Proper immunoprecipitation of pTH2A by both anti-pTH2A Ab1 and Ab2 was also confirmed by western blot analysis (Figs S4a and S5f). Existence of ~150 bp bands corresponding to mono-nucleosomal DNA, as well as to some low-molecular weight DNA (~130 bp and less), was confirmed in inputs (which is Sup in Fig. 3a), and these were equally immunoprecipitated by anti-pTH2A Ab1 and Ab2 (Fig. S4b, upper panel). Single ~280 bp DNA bands were obtained by library preparation (Fig. S4b, lower panel) and sequenced. ChIP reads from replication 1 and 2 correlated significantly with each other (R = 0.890, Fig. S4c). For data analyses, the dataset of ChIP-seq from Erkek *et al*.^33^ containing H3.3, H3.1/3.2, H3K4me3, and H3K27me3 was included as a comparison. The results demonstrated a remarkable enrichment of pTH2A around TSS ±5 kb; the enrichment level was similar to H3.3 and much lower than H3K4me3 (Fig. 3c,d). No preferential distribution of pTH2A among transcriptional ending sites (TES), genic, and intergenic regions was observed (Fig. 3c). Gene ontology (GO) analysis of pTH2A-enriched genes exhibited significant enrichment in GO terms related to development and morphogenesis (system development, anatomical structure morphogenesis, organ development, etc.) and metabolism (regulation of biosynthetic processes, regulation of metabolic processes, etc.; Fig. S4d). RNA metabolism and positive transcriptional regulation, while not GO terms related to reproduction or spermatogenesis, were enriched (Fig. S4d). Heat map analysis to ascertain the co-relationships between pTH2A and H3 variants/modifications in 2,500 randomly selected genes exhibited four distinct clusters as shown in Fig. 3e. It also demonstrated co-enrichment of pTH2A and H3.3 in TSSs in three clusters (Cluster 1, 2, and 3; 1,882/2,500 genes, 75.28%), suggesting a marked overlap between pTH2A and H3.3 in the sperm genome (Fig. 3e). In Cluster 2 and 3, pTH2A and H3.3 were co-localized with H3K4me3 (Fig. 3e, Cluster 2 and 3, 35.16% and 13.56%, respectively). H3K4me3 was more enriched in Cluster 3 than in Cluster 2, while H3.1/2 was excluded from TSSs in both clusters (Fig. 3e). In contrast, in Cluster 1, pTH2A and H3.3 were preferentially co-localized with H3K27me3 rather than H3K4me3 (Fig. 3e, Cluster 1, 26.56%). Cluster 4 (24.72%) exhibited no substantial enrichment in any of the examined histones and modifications. Collectively, our ChIP-seq analyses demonstrated the substantial enrichment of sperm-retained pTH2A around TSSs, and showed that pTH2A behaved similarly to H3.3 when the sperm chromatin was solubilized by high DTT and MNase conditions.

### Paternal pTH2A is removed upon fertilization and recurrent in the first mitosis

**Figure 4:**
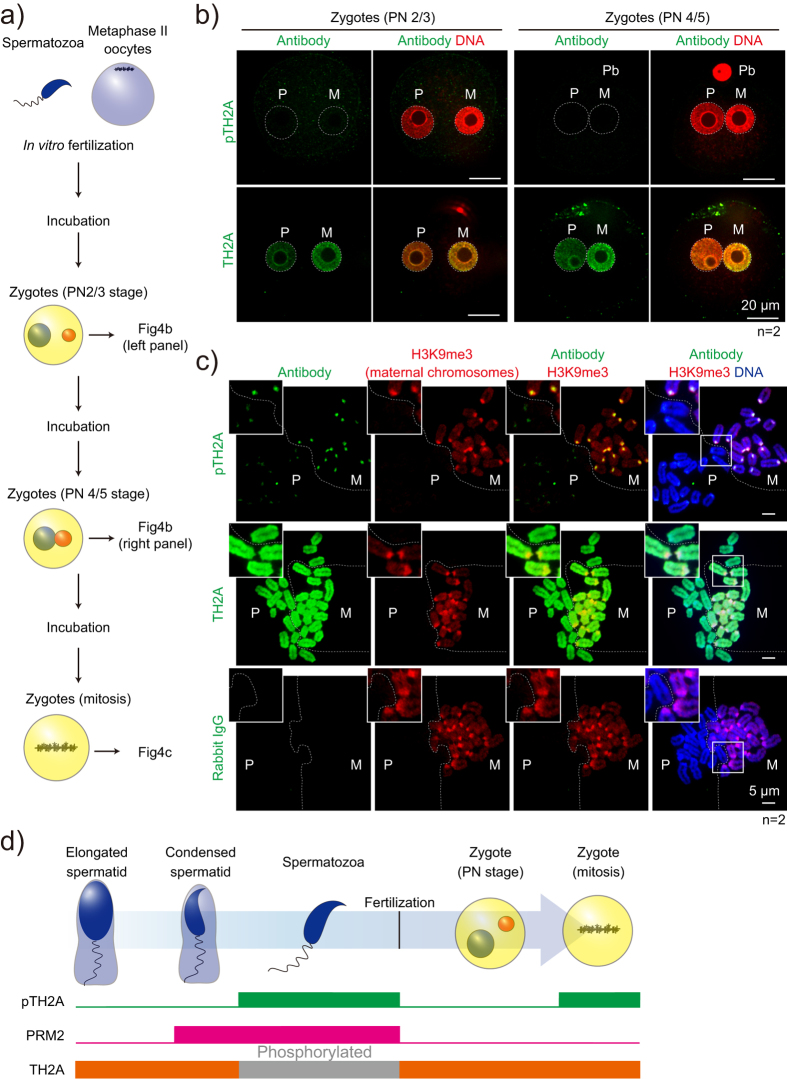
Dynamics of pTH2A after fertilization. (**a**) An experimental schema of the immunofluorescent staining of zygotes. (**b**) Immunofluorescent staining in the PN 2/3 (left panel) and 4/5 (right panel) stage of zygotes. White dashed lines indicate each pronuclei. (**c**) Immunofluorescent staining of mitotic zygotes prepared by chromosome spread. White boxes indicate the magnified area, and white dashed lines separate paternal and maternal chromosomes. Antibodies used and scale bars are as indicated. Number of biological replicates of each experiment is shown as n. Abbreviation: P, paternal; M, maternal; Pb, polar body. (**d**) Schematic summary of pTH2A dynamics across spermiogenesis and 1-cell stage. Presence of pTH2A, PRM2, and TH2A is shown by colored boxes.

Since TH2A and TH2B are maternal-effect proteins and have been reported to play critical roles in paternal zygotic transcription^13^, we next pursued the dynamics of pTH2A in zygotes. Zygotes obtained from *in vitro* fertilization were subjected to immunostaining analyses at pronuclear stage (PN) 2–3, PN4–5, and mitotic phase (Fig. 4a). As previously demonstrated, TH2A was abundantly expressed in both maternal and paternal pronuclei in both PN2–3 and PN4–5 (Fig. 4b)^13^. In contrast, although pTH2A was detected in caudal epididymal spermatozoa by western blotting (Figs 1d,f and 3a), it was absent in the paternal pronucleus in both PN2–3 and PN4–5 zygotes (Fig. 4b). Maternal pronuclei also lacked pTH2A, although the existence of pTH2A in unfertilized oocytes has not been investigated (Fig. 4b). This observation suggests that paternal pTH2A is cancelled at fertilization and does not transfer to the embryos, although GO analyses of pTH2A-enriched genes in the sperm genome exhibited significant enrichment of developmental genes (Fig. S4d). Unexpectedly, pTH2A was recurrent in the first mitotic chromosomes and enriched in pericentromeric heterochromatin, in which H3K9me3 was highly accumulated (Fig. 4c). Interestingly, pTH2A was more abundant in maternal than paternal chromosomes (Fig. 4c), clearly indicating the asymmetrical susceptibility to pTH2A of paternal and maternal chromatin.

In summary of these findings, the present study identified a novel histone phosphorylation in a germ cell-specific H2A variant TH2A, which specifically occurs in condensed spermatids and pericentromeric heterochromatin in mitotic zygotes (Fig. 4d), implying their unique role in chromatin condensation.

## Discussion

The substitution of histones by variants is an enabler of cell type-specific events. Germ cell development is one such instance where histone variants, including both germ cell-specific and ubiquitous types, are intricately involved in corresponding dynamic changes of genome organization such as meiosis and post-meiotic chromatin condensation. Sperm histones and their modifications have recently become the subject of intensive investigation due to their potential importance for transgenerational effects^33^,^34^,^35^. However, identification of variant-specific modifications in histones is technically difficult because of the high similarity of amino acid sequences between variants. In this study, we successfully demonstrated the variant-specific phosphorylation of TH2A at the unique C-terminal tail. We showed that this specifically occurs in condensed spermatids and in the first mitosis of early preimplantation embryos (Figs 1b and 4d), which could not be clarified by a previous search of sperm histone modifications by mass spectrometry^2^.

Histone phosphorylation is involved in various cellular events, such as DNA damage response, transcription, mitosis/meiosis, and apoptosis^36^. Based on the fact that pTH2A is observed in condensed spermatids and the mitotic centromere (Figs 2b and 4c), it is assumed that pTH2A is involved in chromatin condensation, although the structural importance of the C-terminal tail of TH2A in histone-DNA and histone-histone interactions has not been demonstrated^13^,^37^. Interestingly, however, this region was shown to be required for TH2A/TH2B-induced iPSC generation, as a mutant TH2A in which the C-terminal tail was swapped with that of canonical H2A failed to convert fibroblasts to iPSCs^37^. This implies the possibility that pTH2A might be involved in cell reprogramming. In addition, although the C-terminal sequence of human TH2A is not identical to that of mice, the amino acid corresponding to mouse Thr127 in human is Ser129, and surrounding sequences are well-conserved (Figs 1c and S1a), implying that the humanTH2A Ser129 is possibly phosphorylated.

One of the dynamic chromatin events characteristic of germ cells is the histone-protamine exchange required for sperm nuclear condensation, and pTH2A was found to be enriched in this stage as well. In our histological analysis, pTH2A began to be observed in the condensed spermatids of spermatogenic stage III, when the histone-protamine exchange was completed (Figs 2b and S3c,d). It is thus unlikely that pTH2A plays a role in histone removal or protamine incorporation. The behavior of kinase is also intriguing since it is expressed and functions in condensed spermatids; as nucleosomes are highly condensed here, it may be difficult for the kinase to access TH2A. Identification of the kinase will lead to an improved understanding of the structure of sperm histones and the physiological role of pTH2A.

Considering the different MNase sensitivity of TH2A from that of TH2B (Fig. 3a), it is assumed that pTH2A may not preferentially dimerize with TH2B in sperm, although the co-crystal structure of TH2A and TH2B has been previously demonstrated^37^. In fact, TH2B is reported to exist in an alternative chromatin structure, in which other H2A variants (H2AL1/L2) are enriched and associated with <100 bp DNA in condensed spermatids^6^. ChIP-seq analyses demonstrated remarkable overlap between pTH2A and H3.3 in TSS ±5 kb (Fig. 3e). Moreover, pTH2A-enriched genes are related to development and morphogenesis (Fig. S4d), implying the transgenerational effect of paternal pTH2A on embryonic development. However, the specific genomic locations of sperm-retained histones are currently controversial^38^, and the enrichment around TSSs might be a general property of sperm histones intended to provide a more accessible structure necessary for early utilization during embryogenesis^39^. In fact, sperm pTH2A does not persist in paternal pronuclei (Fig. 4b), although it is possible that a small amount of pTH2A is retained in TSSs of such genes after fertilization and effects gene transcription in embryos. Further investigation using pTH2A-deficient mice is required to examine the effects of sperm pTH2A in the next generation.

It is notable that after fertilization, pTH2A is recurrent in the first mitosis and highly enriched in pericentromeric heterochromatin (Fig. 4c). Since pTH2A was not detected in *in vitro*-cultured GS cells and TH2A-overexpressed HEK293T cells, at least in western blot (Fig. 1f), it is unlikely that pTH2A is a general mitotic marker like H3 Ser10 phosphorylation. Thus, one question awaiting further investigation is which a particular kinase is responsible for pTH2A. Also, pTH2A kinase(s) in condensed spermatids and fertilized embryos may not be identical, as in the latter case maternally-derived proteins are functionally dominant. In addition, TH2A conserves threonine at the 120^th^ amino acid and corresponds to H2A-T120, which is phosphorylated during mitosis by Bub1 kinase and is required for proper chromosome segregation^18^. Since the protein expression of Bub1 is maintained in MII oocytes^40^, it is possible that it persists in zygotes and phosphorylates TH2A T120 in the first mitosis. In addition, recent proteomic analyses of mouse testis found that the acetylation of TH2A at the N-terminus is also expected^41^.

In summary, this study discovered a novel, variant-specific histone modification and demonstrated its association with chromatin condensation in a particular set of circumstances *in vivo*. Further functional characterization of pTH2A will aim to uncover its physiological roles in spermiogenesis, early embryonic mitosis, and transcription.

## Methods

### Animal types and ethics statement

Rabbits (Japanese White strain; OrientalBioService, Kyoto, Japan) were used for developing antibodies. All experimental procedures involving immunization of rabbits were approved by the Animal Experiment Management Committee and Animal Experiment Steering Committee at Medical & Biological Laboratories. For mouse experiments, C57BL/6, ICR, BDF1 (C57BL/6 x DBA/2 strain) and a mixture of C57BL/6 and BDF1 mice (CLEA Japan, Tokyo, Japan) were used for all experiments. All experimental procedures involving mice were approved by the Animal Experiment Ethics Committees at the Institute of Molecular and Cellular Biosciences, University of Tokyo (Exp # 2611, 2710). Experiments were performed in precise accordance with the manual provided by the Life Science Research Ethics and Safety Committee, University of Tokyo.

### Antibodies

All antibodies used in this study are listed in Supplementary Table 1 together with their working concentrations.

### Cell culture and DNA transfection

HEK293T cells obtained from ATCC (ATCC number, CRL-11268) were maintained in high-glucose D-MEM (Wako, Osaka, Japan) containing 10% fetal bovine serum (FBS; BioWest, Nuaillé, France), 1% GlutaMAX (Thermo Fisher Scientific, Waltham, MA, USA), and 1% penicillin/streptomycin (Thermo Fisher Scientific). Isolation and *in-vitro* culture of germ stem (GS) cells were performed as described previously^42^,^43^. Cells were maintained at 37 °C in 5% CO_2_. We used Lipofectamine 2000 reagent (Invitrogen, Carlsbad, CA, USA) for the transfection of FLAG-tagged H2A and FLAG-tagged TH2A into HEK293T cells, according to the manufacturer’s instructions.

### Alignment of amino acid sequence

Amino acid sequences of mouse H2A type1 (NP_835495), mouse TH2A (NP_783589), human TH2A (NP_734466), and rat TH2A (NP_068611) were obtained from the NCBI database. These sequences were loaded into ClustalX2 software^44^ and aligned using the default setting. Sequences then were loaded into GeneDoc2.7 software^45^ with default settings and used to create aligned images (Fig. S1a). The percentage of the sequence homology of mouse TH2A against rat TH2A and human TH2A was calculated using GENETYX Ver.12.0.4 software (Genetyx, Tokyo, Japan) using the default parameters.

### Generation of anti-pTH2A antibody

Anti-pTH2A antibodies were generated at Medical and Biological Laboratories (MBL; Nagoya, Japan). Synthesized mouse pTH2A peptide (CKTESHKSQphoTK; Fig. 1c) was used as an antigen. The synthesized peptide was conjugated to keyhole limpet hemocyanin (KLH) and immunized to two Japanese white rabbits. After four immunizations over two months, the antiserum from each rabbit was purified by loading onto a column filled with pTH2A peptide-binding beads. To eliminate cross-reactivity with non-phosphorylated TH2A, the affinity-purified polyclonal antibody was further absorbed by using the non-phosphorylated TH2A peptide (CKTESHKSQTK). The reactivity of the purified antibodies to the pTH2A peptide was confirmed by using an ELISA plate coated with pTH2A peptide or non-phosphorylated TH2A peptide (Fig. S2a–d). Briefly, 96-well ELISA plates (Nunc, Roskilde, Denmark) were coated with 100 μl/well of the immunized peptide or non-phosphorylated TH2A peptide as a negative control antigen at 4 °C overnight. Then, the ELISA plates were washed with phosphate buffer saline (PBS) once and blocked with 200 μl/well of blocking solution containing 5% bovine serum albumin (BSA) overnight at 4 °C. After discarding the blocking solution, the collected rabbit antisera were added to the ELISA plate and incubated for 1 hour at 25 °C. After washing four times with PBS containing 0.05% Tween20, the plates were reacted with 100 μl/well of horseradish peroxidase (HRP)-conjugated goat anti-rabbit IgG (MBL) for 1 hour at 25 °C. After washing the plates with 0.05% Tween20/PBS four times, tetramethylbenzidine was added as a substrate for HRP. The reaction was stopped with 2N H_2_SO_4_, and OD value at 450 nm was measured with a microplate reader (Tecan, Männedorf, Switzerland).

### Peptide blocking assay

Anti-pTH2A antibodies were mixed with 5 μg/mL of unmodified (KTESHKSQTK) or phosphorylated (KTESHKSQphoTK) TH2A peptides, then used as primary antibody solution. The detailed methods of western blot analysis were described below.

### Nucleosome extraction from mouse spermatozoa

Extraction of sperm histones was performed by following the protocol published by Hisano *et al*.^32^ with slight modifications. Male mice aged 8–12 weeks were sacrificed by cervical dislocation and their cauda epididymis were isolated. Approximately 4 × 10^7^ spermatozoa collected from the cauda epididymis were suspended in a cytoplasmic lysis buffer (25 mM Tris-HCl pH 7.5, 150 mM NaCl, 0.1% Nonidet P-40 (NP-40)). After centrifugation for 2 min at 20,400 *g* at 4 °C, cell pellets were incubated in 150 μl of sperm lysis buffer (50 mM Tris-HCl pH 7.4, 150 mM NaCl, 1% NP-40, 0.5% sodium deoxycholate, 0.2% SDS, 4.5 mM sodium acetate, 20 mM DTT, Complete Protease Inhibitor Cocktail Tablets (Roche, Basel, Switzerland)) for 10 min on ice. After centrifugation for 10 min at 20,400 *g* at 4 °C, the supernatant was used as the sperm S1 fraction. The chromatin pellet was incubated in the same quantity of sperm lysis buffer and MNase digestion buffer (10 mM Tris-HCl pH7.4, 10 mM KCl, 5 mM CaCl2, 4.5 mM sodium acetate, 20 mM DTT, Complete Protease Inhibitor Cocktail Tablets; total volume 300 μl) with 0.3 unit/μl MNase at 37 °C for 30 min. Digestion was stopped by adding ethylenediaminetetraacetic acid (EDTA) pH 8.0 to a final concentration of 18 mM. The sperm S2 fraction was isolated by centrifugation for 10 min at 20,400 *g* at 4 °C, and the pellet was used as the sperm Ppt fraction (Fig. 1a).

### λPPase treatment of sperm S2 fraction

λPPase and supplied reagents (New England Biolabs, Ipswich, MA, USA) were added to the sperm S2 fraction according to manufacturer’s instruction. Reaction was performed by incubation in 37 °C for 30 min, then stopped by adding 2× SDS sample buffer (100 mM Tris-HCl pH6.8, 4% SDS, 0.02% bromophenol blue, 20% glycerol, 10% 2-mercaptoethanol).

### In-gel digestion of sperm histones

In-gel digestion of sperm histones was performed as described previously, with some modifications^46^. The sperm S2 fraction was resolved by Any kD Mini-PROTEAN TGX Precast Protein Gels (Bio-Rad, Hercules, CA, USA), then visualized using a Silver Stain Kit (Bio-Rad). For in-gel digestion of sperm histones, a One step CBB stain kit (Bio Craft, Tokyo, Japan) was used for visualization, and gels from 10–20 kDa, which were considered to contain histone proteins except for histone H1, were excised. These gels were washed with first destaining solution (25 mM ammonium bicarbonate (ABC), 50% acetonitrile (ACN)) and second destaining solution (25 mM ABC, 30% ACN), then incubated in 100% ACN for 15 min. Gel pieces were dried using a centrifugal concentrator (Taitec, Saitama, Japan), then re-swelled with 0.8 ng/μl ArgC (Roche), prepared according to the manufacturer’s instructions. Digestion by ArgC was carried out for 16 h at 37 °C. The supernatant was transferred into new tubes, and residual gel pieces were extracted with an elution solution (5% formic acid, 50% ACN) under constant shaking for 20 min. This step was performed four times. The collected supernatant was dried completely using a centrifugal concentrator and resolved with transport buffer (0.1% formic acid, 2% ACN).

### Mass spectrometric analysis

Nano liquid chromatography tandem mass spectrometry (Nano-LC-ESI-MS/MS) analysis was performed as described previously, with some modifications^46^. Briefly, peptides were analyzed using a DiNa-2A system (KYA Technologies, Tokyo, Japan) and a LTQ Velos Orbitrap ETD instrument (Thermo Fisher Scientific). The gradient consisted of A solution (A; 0.1% formic acid, 2% ACN) and B solution (B; 0.1% formic acid, 80% ACN) in the following sequence: 0–50% B from 0–100 min, 100% B from 100–110 min, and 0% B from 110–120 min. Flow rate was a constant 300 nl/min. MS spectra were recorded for *m/z* 450–2000 at a resolution of 60,000, followed by data-dependent collision-induced dissociation MS/MS spectra and electron transfer dissociation MS/MS spectra generated from the five highest-intensity precursor ions. The MS/MS data were analyzed using Proteome Discoverer Version 1.3 (Thermo Fisher Scientific) against SEQUEST and ZCore (Thermo Fisher Scientific), and evaluated against a cutoff of a 5% false positive rate. Phosphorylated serine and threonine were set as dynamic modifications.

### Preparation of cell extracts

HEK293T cells expressing FLAG-tagged H2A, and FLAG-tagged TH2A *in vitro*-cultured GS cells, were washed with PBS, and cell pellets were collected by centrifugation at 600 *g* for 5 min. Cells were resolved in lysis buffer (25 mM Tris-HCl pH 7.5, 500 mM NaCl, 0.1% NP-40) and pelleted by centrifugation at 20,400 *g* for 1  min. These pellets were incubated in 1× SDS sample buffer at 98 °C for 15 min for complete dissolution. Whole testicular cells were dispersed by trypsin/EDTA at 37 °C for 15 min, and trypsin activity was stopped by adding FBS. Cell suspension was filtered through a 70 μm cell strainer (Becton Dickinson, Franklin Lakes, NJ, USA), and collected by centrifugation at 600 *g* for 5 min. Cell pellets were incubated in 1× SDS sample buffer at 98 °C for 15 min. These cell extracts and the sperm S2 fraction were analyzed by western blot (Fig. 1f).

### Western blot

We used two kinds of detection system for western blot analysis: an Odyssey infrared imaging system (LI-COR, Lincoln, NE, USA) and a LAS chemiluminescence system. The applications for each antibody are indicated in Fig. S5, which includes the raw images. Protein samples were resolved with 15% of SDS-polyacrylamide gel for 100 min at 110 volts at room temperature, then transferred to Immobilon-FL (for Fig. 1a,e,f) or -PSQ membrane (for others) (Merck Millipore, Billerica, MA, USA) for 1 hour at 90 volts. In the Odyssey imaging system, membranes were blocked using Odyssey Blocking Buffer (LI-COR) for 30 min, then incubated with primary antibodies (Table S1) diluted in Odyssey Blocking Buffer containing 0.2% Tween 20 overnight. Membranes were incubated with IRDye 800CW Donkey anti-Rabbit IgG (1:15,000, LI-COR) diluted in Odyssey Blocking Buffer containing 0.2% Tween 20 and 0.01% SDS. The signals were detected by an Odyssey infrared imaging system, with detection intensity programmed in Image Studio 2.0 (LI-COR), as shown in Fig. S5. Acquired images were processed by Image Studio 2.0. In the LAS chemiluminescence system, membranes were blocked using 5% BSA in TBS-T (for H3K27me3 and H2A) or Odyssey Blocking Buffer containing 0.2% Tween 20 (for others) for 30 min. These were then incubated with primary antibodies diluted in MAX blot (MBL) or Odyssey Blocking Buffer containing 0.2% Tween 20, respectively, overnight. The signals were detected using Donkey anti-rabbit IgG conjugated to HRP (1:5,000, GE, Illinois, USA) and the LAS 4000 (Fujifilm, Tokyo, Japan); exposer time is indicated in Fig. S5. Acquired images were processed by ImageJ software (National Institute of Health, Bethesda, Maryland, USA). For the detection of pTH2A and TH2A in the same experiment, the membranes were prepared independently for both antibodies and not reprobed, as both antibodies were generated from rabbit.

### Immunostaining of frozen testis sections

Dissected testes were fixed with 4% paraformaldehyde (PFA) and dehydrated using 10%, 20%, and 30% sucrose in PBS, then embedded in an optimal cutting temperature compound (Sakura Finetech, Tokyo, Japan). Sliced sections of 15 μm were air-dried and washed with TBST. Antigen retrieval was achieved by incubation in sub-boiled citrate solution (10 mM sodium citrate pH 6.0, 0.1% Tween-20) in a microwave oven. These sections were blocked with a blocking buffer (5% goat serum (Vector Laboratories, Burlingame, CA, USA) in TBST), and incubated with primary antibodies (Table S1) diluted in blocking buffer. After washing, the slides were incubated with Goat anti-Rabbit IgG (H + L) Secondary Antibody, Alexa Fluor 594 (1:500, Thermo Fisher Scientific), fluorescein isothiocyanate-conjugated PNA (2 ng/μl; Sigma Japan, Tokyo, Japan), and Hoechst 33342 (20 ng/μl, Invitrogen). The images were acquired using a FV1200 or 3000 confocal laser scanning microscope (Olympus, Tokyo, Japan). For image acquisition, “serial acquisition mode” was used to prevent any non-specific leakage of fluorescence, and all microscope/laser settings were identical between samples that were to be compared. More detailed settings of microscope/software are available upon request. Data were processed using ImageJ software; imaging parameters are summarized in Supplementary Table 4.

### Whole-mount Immunostaining of zygotes

MII-stage oocytes were collected from superovulated female mice that were prepared by injecting 7.5 IU pregnant mare serum gonadotropin (ASKA Pharmaceutical, Tokyo, Japan) and 7.5 IU human chorionic gonadotropin (ASKA). Spermatozoa collected from cauda epididymis were incubated for 1 h at 37 °C, 5% CO_2_ in TYH medium (LSI Medience, Tokyo, Japan) to achieve capacitation. MII oocytes were fertilized by *in vitro* fertilization, during which about 2–4 × 10^5^ capacitated sperm were added in HTF medium (Ark Resources, Kumamoto, Japan). At 4 h post insemination, zygotes were collected and further incubated until they reached pronuclear 2/3 or 4/5 stages. Zygotes in each stage were fixed in 3.6% PFA in PBS containing 0.2% Triton X-100 for 20 min, then washed by TBS containing 0.1% Triton X-100. Zygotes were incubated in 5% goat serum diluted TBS containing 0.1% Triton X-100 for 1 h, then treated with primary antibodies (Table S1) diluted with dilution buffer (5% goat serum in TBS containing 0.01% Triton X-100). After washing with dilution buffer, zygotes were incubated with Goat anti-Rabbit IgG (H + L) Secondary Antibody, Alexa Fluor 594 (1:500). The zygotes were then mounted in Vectashield anti-bleaching solution with 4’,6-diamidino-2-phenylindole (DAPI, Vector Laboratories). Images were acquired using a CV1000 Confocal Scanner System (Yokogawa, Tokyo, Japan) and data were processed using ImageJ software. Imaging parameters are summarized in Supplementary Table 4.

### Chromosome spread of zygotes

Chromosome spread of zygotes was performed as described previously^47^. Zygotes with two distinct pronuclei were collected and further incubated for 5–6 hours, then transferred into HTF medium containing 100 ng/ml nocodazole (Sigma Japan) overnight. The zygotes were then transferred into HTF medium containing 1 μM MG-132 (Peptide institute, Osaka, Japan) for 30 min^48^. Zona pellucida were removed using Tyrode’s solution (Sigma Japan), then zygotes were spotted onto MAS-coated slide glass (Matsunami Glass, Osaka, Japan) in fixative solution (1% PFA, 0.15% Triton X-100, 3 mM DTT in distilled H_2_O adjusted to pH 9.2). After a quick dry, the slides were washed with TBS and blocked with blocking buffer (3% BSA in TBS), then incubated with primary antibodies (Table S1) that were diluted with blocking buffer. After washing with TBS, the slides were incubated with Donkey anti-Rabbit IgG (H + L) Secondary Antibody, Alexa Fluor 488 (1:500, Thermo Fisher Scientific), Donkey anti-Mouse IgG Secondary Antibody, Alexa Fluor 568 (1:500), and Hoechst 33342 (20 ng/μl). Images were acquired using an IX-83 inverted fluorescent microscope (Olympus, Tokyo, Japan) and data were processed using ImageJ software. Imaging parameters are summarized in Supplementary Table 4.

### Native chromatin immunoprecipitation (ChIP) from sperm nucleosomes

An aliquot of the sperm S2 fraction described above was kept for input control. Anti-pTH2A antibodies were bound to Dynabeads Protein G (Thermo Fisher Scientific), then mixed with the sperm S2 fraction. Immunoprecipitates were washed with low salt buffer (20 mM Tris-HCl pH 8.1, 150 mM NaCl, 2 mM EDTA, 1% Triton X-100, 0.1% SDS) once, high salt buffer (20 mM Tris-HCl pH 8.1, 500 mM NaCl, 2 mM EDTA, 1% Triton X-100, 0.1% SDS) twice, LiCl buffer (20 mM Hepes-OH pH 7.4, 500 mM LiCl, 1 mM EDTA, 0.5% deoxycholate, 1% NP-40) four times, and TE 50 buffer (50 mM Tris-HCl pH 8.1, 10 mM EDTA) once sequentially, then eluted with elution buffer (1% SDS in TE 50 buffer) at 65 °C for 20 min. The eluates were incubated at 65 °C overnight, then the RNAs were degraded with RNase A (Sigma Japan) at 50 °C for 1 h. Proteins were degraded with Proteinase K at 50 °C for 2 h. Immunoprecipitated DNA was purified using QIAquick PCR Purification kit (Qiagen, Hilden, Germany). The quality of DNA was checked using a High Sensitivity Chip on the Agilent Bioanalyzer 2100 (Agilent Technologies) according to manufacturer’s instruction.

### Sequencing and data processing

High-throughput sequencing was performed using a Hiseq 2000 system (Illumina, San Diego, CA, USA) according to manufacturer’s instructions. ChIP-seq libraries were generated using NEBNext ChIP-Seq Library Prep Master Mix Set for Illumina (New England Biolabs) according to manufacturer’s instruction. Briefly, ChIPed DNAs were ligated to sequencing adapters, and DNA of 200–300 bp were size-selected using AMPure XP (Beckman Coulter, Brea, CA, USA). Amplified libraries were sequenced to generate single-end 51-bp reads. Information and statistics regarding high-throughput sequencing as well as their mapping rates are summarized in Supplementary Table 2. Data sets of H3.3 (GSM1046829), H3.1/H3.2 (GSM1046830), H3K4me3 (GSM1046832), and H3K27me3 (GSM1046835) were obtained from Erkek *et al*.^33^. For control, sperm sonicated genomic DNA (GSM1046836) was used according to the author’s methods (GEO accession number: GSE42629). Sequenced reads were aligned to the mouse reference sequence (mm10) using Bowtie2 v2.1.0 with default parameters^49^. Mapped reads in a 100-bp bin were summed along each chromosome, followed by filtration of duplicate reads and normalization with the total mapped read number by DROMPA2 v2.5.1^50^. Data reproducibility was measured by calculating ChIP read intensities ±1 kbp around pTH2A peaks identified by DROMPA2 with the “-pthre1 5e-2 -qthre 5e-2” option. Calculated read intensities are shown as a smoothed scatter plot (Fig. S4c). The mapped read numbers on TSS, TES, genic and intergenic regions, as well as the averaged ChIP enrichments with a 95% confidence interval around TSS, were calculated by an in-house pipeline. For heat-map plots of ChIP read distributions around TSS, values were scaled from 0 to 1 within each data set. Of the protein-coding genes, 2,500 genes were randomly selected for visualization. Each gene was then grouped by hierarchical clustering with Ward’s method using scaled read intensities ±1 kbp around TSS using the core function hclust in R^51^. Genes were divided into four clusters, the empirically selected minimal number that resulted in distinct clusters. Genes were sorted by the average read density of pTH2A rep1 around TSSs (±1 kbp). Datasets from this study have been deposited in the NCBI Sequence Read Archive (SRA) under accession number SRP091482.

## Additional Information

**How to cite this article:** Hada, M. *et al*. Identification of a variant-specific phosphorylation of TH2A during spermiogenesis. *Sci. Rep.* **7**, 46228; doi: 10.1038/srep46228 (2017).

**Publisher's note:** Springer Nature remains neutral with regard to jurisdictional claims in published maps and institutional affiliations.

## Supplementary Material

Supplementary Information

Supplementary Table S1

Supplementary Table S2

Supplementary Table S3

Supplementary Table S4
