# Supplementary material for: Identification of a variant-specific phosphorylation of TH2A during spermiogenesis
Source: Sci Rep. 2017 Apr 7;7:46228. doi: 10.1038/srep46228 (PMC5384234; doi:10.1038/srep46228)

Identification of a variant-specific phosphorylation of TH2A during spermiogenesis

Masashi Hada[1,3], Koji Masuda[2], Kosuke Yamaguchi[1,4], Katsuhiko Shirahige[2], and Yuki Okada[1]

Supplementary methods

Plasmid construction

Total RNA was isolated from mouse testis using NucleoSpin RNA (Takara, Shiga, Japan) and reverse transcribed by SuperScript III (Invitrogen, Carlsbad, CA, Japan). Generated cDNAs were amplified by Prime STAR MAX (Takara) with following primers:

mouse H2A-forward, 5’-gggCTCGAGatgtctggacgcggaaagcagg-3’;

mouse H2A-reverse, 5’- aaaGCGGCCGCTTACTTCCCCTTGGCCTTGTGG-3’;

mouse TH2A-forward, 5’- gggCTCGAGatgtctggtcctacaaagcgggg-3’;

mouse TH2A-reverse, 5’-aaaGCGGCCGCTCACTTGGTCTGGGACTTGTGG-3’.

These products were inserted into pMSCV vector harboring FLAG-tag in its N-terminus.

Gene ontology analysis

Gene ontology analysis of pTH2A-enriched genes was performed using the DAVID gene functional classification tool[1] . Mapped reads of input and ChIPed DNA against pTH2A Replicate1 around 1 kbp from TSS were summed and normalized for the total number of mapped reads (Table S3). Genes in which the normalized number of input was above 0.5 ppm were extracted. Enrichment of pTH2A was calculated and pTH2A-enriched genes (enrichment > 2.0) were subjected to gene ontology analysis with default parameters.

Supplementary figure legends

Figure S1. Alignment of the amino acid sequences of TH2A between mouse, rat, and human detection of H4 acetylation and H4K20 methylation in sperm chromatin and

(a) Amino acid sequences of TH2A in mouse (NP_783589), rat (NP_068611), and human (NP_734466) were obtained from the NCBI database. Sequence homology from mouse to rat or human is shown as a percentage. Representative MS/MS spectra for (b) H4K5, 8, 12, 16 acetylation and (c) H4K20 methylation detected in the sperm S2 fraction are shown.

Figure S2. Validation of the specificity of anti-pTH2A antibody

(a-d) Affinity of each antibody for (a, c) phosphorylated and (b, c) unmodified peptides was tested by ELISA. Antibodies used are indicated.

Figure S3. Immunostaining analyses of seminiferous tubules using rabbit IgG, α-pTH2A, α-TH2A, and α-PRM2 antibodies. (a) Negative control of immunostaining using IgG instead of primary antibodies corresponding to Fig. 2. (b) Comparison of immunostaining signals of pTH2A, TH2A, and IgG in Stage I and IV-V seminiferous tubules. Detailed image acquisition settings are described in Table S4. (c, d) Incorporation of pTH2A (c) and PRM2 (d) in Stage II and III seminiferous tubules. White boxes indicate magnified areas. Antibodies used and scale bars are as indicated. The number of biological replicates of each experiment is shown as n.

Figure S4. ChIP-seq analysis of pTH2A in sperm chromatin

(a) Western blot analysis of ChIPed-sperm nucleosome against pTH2A (upper panel) and TH2A (lower panel). Two distinct anti-pTH2A antibodies (Ab 1 and 2) were used for

immunoprecipitation. (b) Quality check of ChIPed DNA and ChIP-seq library. ChIPed DNA (upper panels) and ChIP-seq library (lower panels) from pTH2A rep1 (left panels) and rep2 (right panels) were analyzed by an Agilent 2100 bioanalyzer. (d) Gene ontology analysis for pTH2A-enriched genes. Top 20 terms of BP3 are listed together with negative log10 of *p*-value. Terms related to development are printed in red.

Figure S5. Uncropped images of silver staining and western blot analysis

Images related to Fig1a (a), Fig1d (b), Fig1e (c), Fig1f (d), Fig3a (e), and FigS4a (f) are shown. The cropped area is shown by a red dashed box. Each intensity (Odyssey inferred system) and exposure time (LAS chemiluminescent system) is indicated.

Reference

1 Huang, D. W. *et al.* The DAVID Gene Functional Classification Tool: a novel biological module-centric algorithm to functionally analyze large gene lists. *Genome Biol.* **8**, 16, doi:10.1186/gb-2007-8-9-r183 (2007).

Figure-S1 (Hada)

a)

```
TH2A
                   20                  40                  60
Mouse:         SGPTKRGGKARAKVKSRSSRAGLQFPVGRVHRLLRQGNYAQRIGAGAPVYLAAVLEYLTAEVLEL
Rat (92.9%):   SGRAKQGGKARAKAKSRSFRAGLQFPVGRVHRLLRQGNYAERIGAGTPVYLAAVLEYLTAEILEL
Human (87.5%): SGRGKQGGKARAKSKSRSSRAGLQFPVGRIHRLLRKGNYAERIGAGAPVYLAAVLEYLTAEILEL

                            80                  100                 120
Mouse:         AGNAARDNKKTRITPRHLQLAIRNDEELNKLLGRVTIAQGGVLPNIQAVLLPKKTES--HKSQTK*:128
Rat (92.9%):   AGNAARDNKKTRIIPRHLQLAIRNDEELNKLLGRVTIAQGGVLPNIQAVLLPKKTES-HHKSQTK*:129
Human (87.5%): AGNASRDNKKTRIIPRHLQLAIRNDEELNKLLGGVTIAQGGVLPNIQAVLLPKKTESHHHKAQSK*:130
```

b)

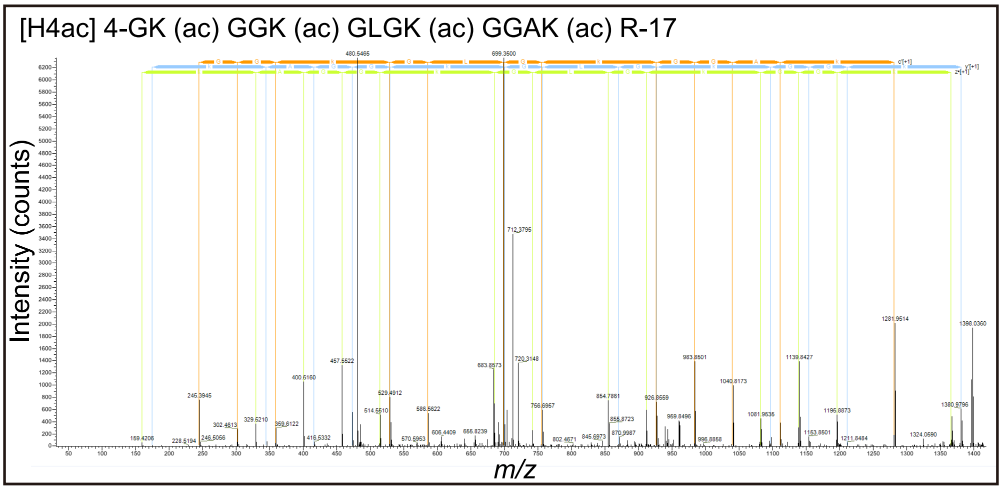

c)

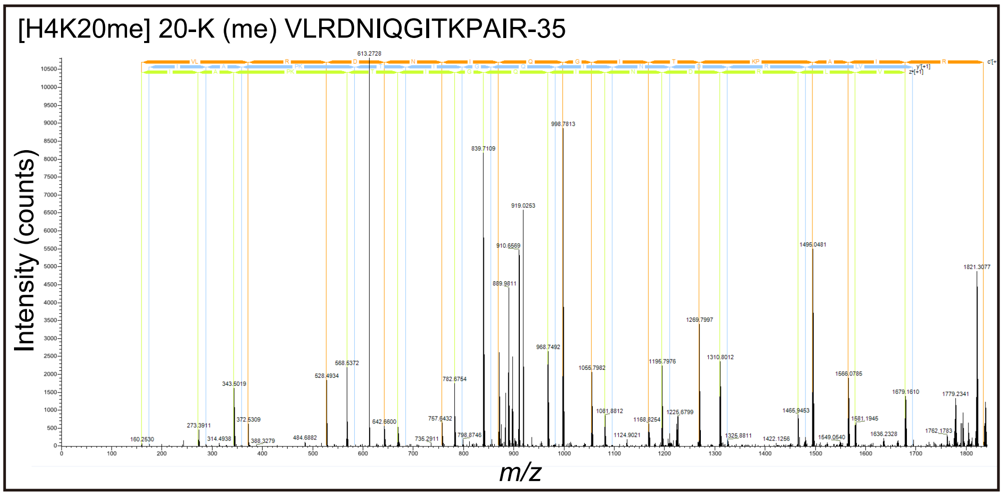

Figure-S2 (Hada)

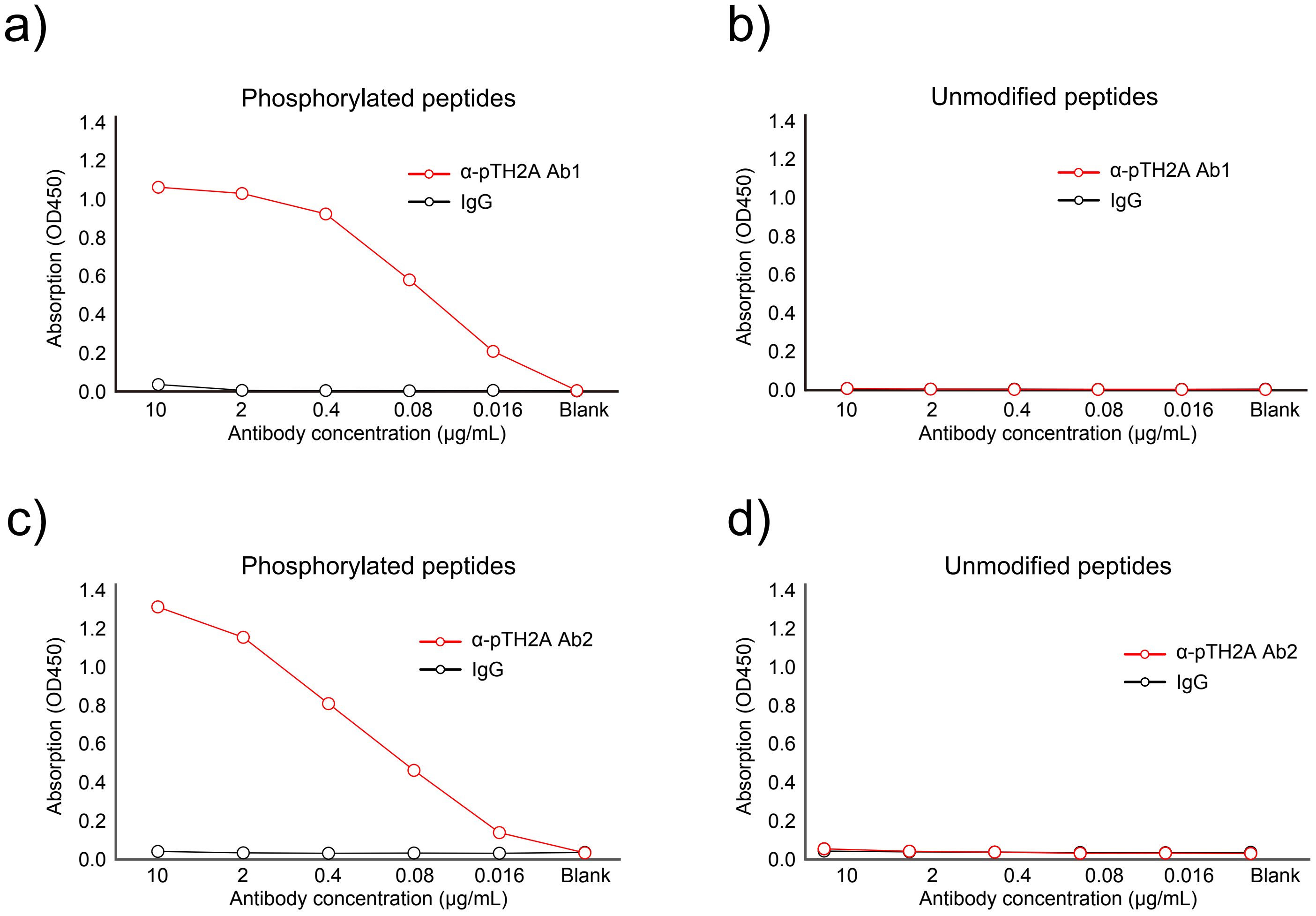

Figure-S3 (Hada)

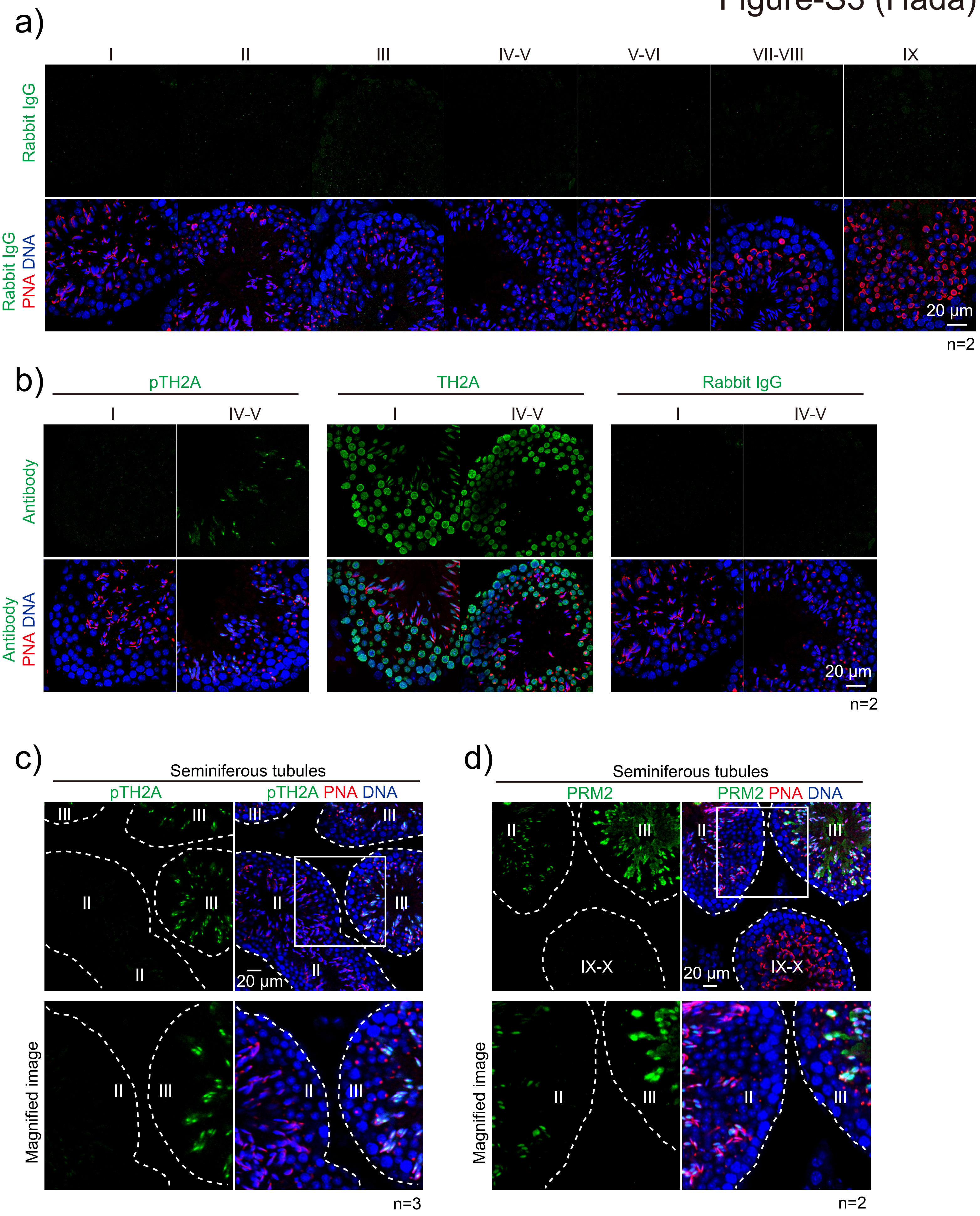

Figure-S4 (Hada)

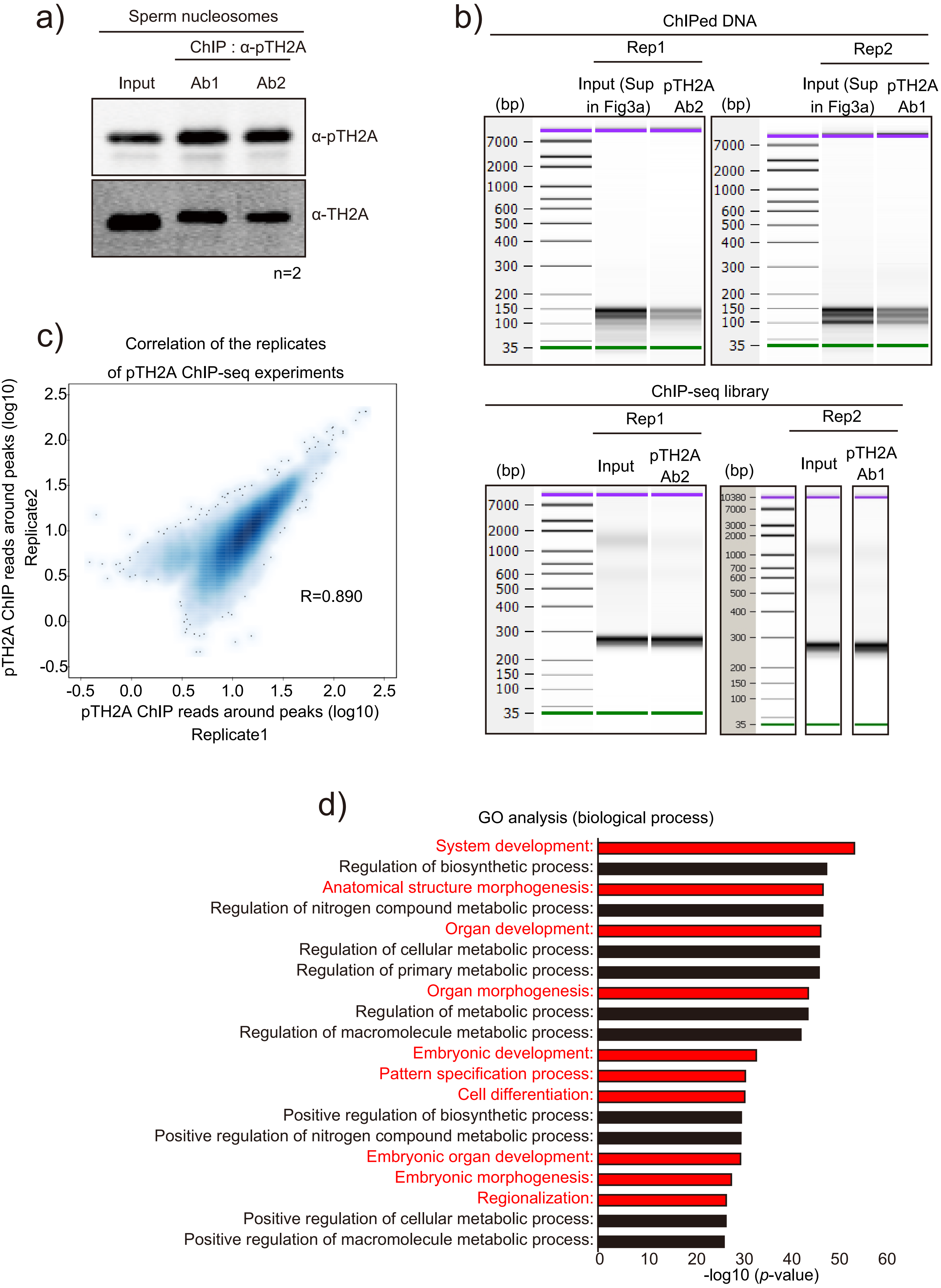

Figure-S5 (Hada)

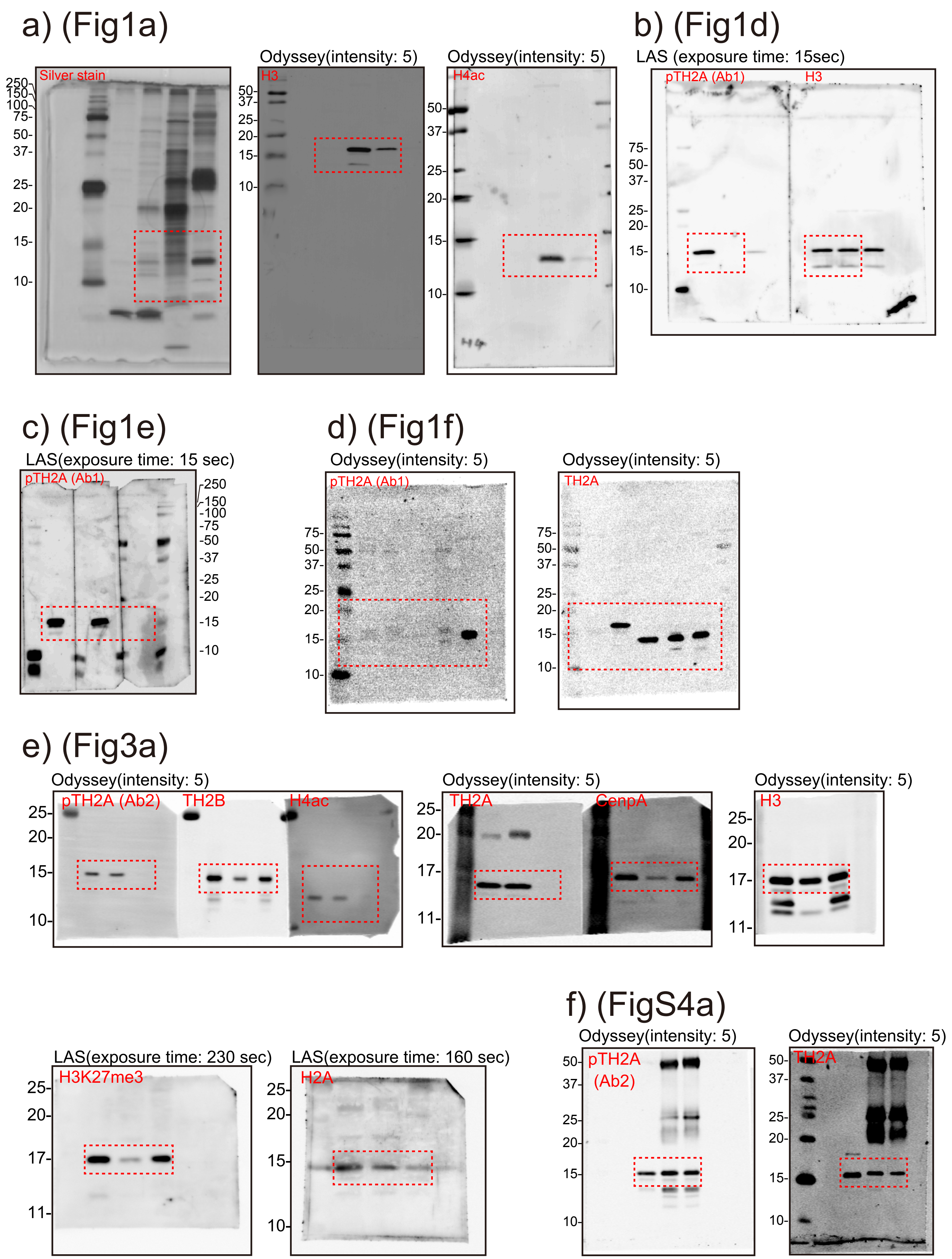

Supplement: Supplementary Information [file srep46228-s1.pdf]
